# Progranulin in frontotemporal lobar degeneration and neuroinflammation

**DOI:** 10.1186/1742-2094-4-7

**Published:** 2007-02-11

**Authors:** Zeshan Ahmed, Ian RA Mackenzie, Michael L Hutton, Dennis W Dickson

**Affiliations:** 1 Department of Neuroscience, Mayo Clinic College of Medicine, Jacksonville, FL, USA; 2 Department of Pathology, University of British Columbia, Vancouver, BC, Canada

## Abstract

Progranulin (PGRN) is a pleiotropic protein that has gained the attention of the neuroscience community with recent discoveries of mutations in the gene for PGRN that cause frontotemporal lobar degeneration (FTLD). Pathogenic mutations in *PGRN *result in null alleles, and the disease is likely the result of haploinsufficiency. Little is known about the normal function of PGRN in the central nervous system apart from a role in brain development. It is expressed by microglia and neurons. In the periphery, PGRN is involved in wound repair and inflammation. High PGRN expression has been associated with more aggressive growth of various tumors. The properties of full length PGRN are distinct from those of proteolytically derived peptides, referred to as granulins (GRNs). While PGRN has trophic properties, GRNs are more akin to inflammatory mediators such as cytokines. Loss of the neurotrophic properties of PGRN may play a role in selective neuronal degeneration in FTLD, but neuroinflammation may also be important. Gene expression studies suggest that *PGRN *is up-regulated in a variety of neuroinflammatory conditions, and increased *PGRN *expression by microglia may play a pivotal role in the response to brain injury, neuroinflammation and neurodegeneration.

## Background

**Figure 1:**
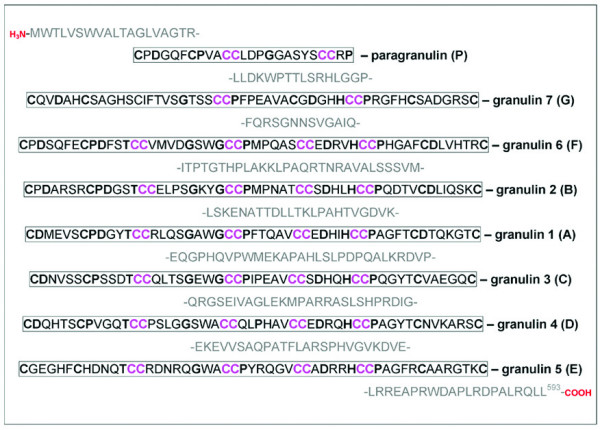
The protein sequence of full length PGRN and its proteolytically cleaved GRNs. Amino acids shown in bold represent the granulin consensus sequence separated by variably long linker regions. Cysteine-rich parts of the sequence are denoted by CC.

Progranulin (PGRN) was discovered independently by several investigators and given several different names, including granulin-epithelin precursor, proepithelin, prostate cancer (PC) cell derived growth factor and acrogranin [1]. Encoded by a single gene on chromosome 17q21 (*PGRN*), PGRN is a 593-amino acid, cysteine-rich protein with an estimated molecular weight of 68.5 kDa that runs at 90 kDa on standard western blots due to heavy glycosylation [2]. It contains seven granulin-like domains, which consist of highly conserved tandem repeats of a rare 12 cysteinyl motif [3,4] (Figure 1). Proteolytic cleavage of the precursor protein by extracellular proteases, such as elastase, gives rise to smaller peptide fragments termed granulins (GRNs) or epithelins [1]. These fragments range in size from 6 to 25 kDa and have been implicated in a range of biological functions [1,5].

Previous work on PGRN focused on its role in embryonic development and neoplasia (reviewed elsewhere [1]). The recent discovery that mutations in *PGRN *cause frontotemporal lobar degeneration with ubiquitin-immunoreactive neuronal inclusions (FTLD-U) has brought renewed interest in PGRN and its functions in the central nervous system (CNS). We review what is known about PGRN in peripheral tissues during injury, repair and inflammation and explore the relevance of these properties to CNS disorders, with a focus on FTLD-U.

## PGRN in the periphery

### Gene expression studies

Basal gene expression studies in mice and rats reveal widespread expression of *PGRN *in many different tissues, as well as in epithelial and hematopoietic cell lines [6,7]. Expression of *PGRN *mRNA is particularly high in epithelial cells that have a rapid turnover, such as those of the skin and gastrointestinal tract. Non-proliferating epithelia, such as lung alveolar cells, have relatively low levels of expression [6]. Epididymal cells have high *PGRN *expression, but are mitogenically stable, implying a pleiotropic role for PGRN. Mesenchymal tissues that lack *PGRN *mRNA are mitogenically responsive to PGRN *in vitro*. Both full length PGRN [1] and its proteolytic peptides [8] have mitogenic affects on epithelial cells in culture. Many transformed or immortalized epithelial cell lines express *PGRN *[3], while primary cells and cells *in vivo *have relatively low *PGRN *expression [6].

PGRN peptides were originally isolated and characterized in activated leukocytes [9]. In the periphery *PGRN *mRNA is abundant in lymphoid tissue of the lung, gut and spleen, and expression is also high in hematopoietic cell lines [6]. Hematopoietic myeloid cells, such as macrophages and tissue histiocytes in liver, spleen, lungs and brain, show no labeling by *in situ *hybridization even though a profile of human macrophages transcripts *in vitro *identified *PGRN *as one of the most highly expressed mRNAs [10]. All *in vitro *results suggests that there are high levels of expression in hematopoietic myeloid cells in the periphery, but low basal level of expression *in vivo*. In contrast to the mitogenic properties of PGRN on epithelial cells, there is little evidence to suggest that PGRN has mitogenic effects on hematopoietic cells [6,11].

### PGRN in wound healing

PGRN is an important growth factor in the wound healing response [12], which can be separated into the distinct phases of inflammation, epithelialization, granulation, neovascularization and contraction (reviewed in [13]). In experimental skin wounds of mice, *PGRN *mRNA increases in fibroblasts, endothelial cells, macrophages and neutrophils. Fibroblasts and endothelial cells have no *PGRN *expression in normal skin, but there is constitutive expression of *PGRN *in keratinocytes. Addition of PGRN to the wound increases and prolongs infiltration of neutrophils and macrophages, and it enhances neovascularization [12], but it has no effect on the overall rate of healing [14]. The role of PGRN in the later stages of wound healing is minimal. Stimulation of fibroblasts and endothelial cells *in vitro *with PGRN causes proliferation and migration, suggesting that injury-induced expression of PGRN may have a paracrine effect. Supporting this hypothesis, PGRN was shown to have the same properties as known stimulators of neovascularization, such as vascular endothelial growth factor, in a cell culture model [12].

### PGRN and inflammation

The wound repair response also sheds light on the roles of PGRN and GRNs during inflammation. Zhu and coworkers demonstrated an immunoregulatory role of PGRN and GRN peptides during wound healing and highlighted novel interactions between PGRN, secretory leukocyte protease inhibitor (SLPI) and elastase. SLPI is a 14-kDa protein encoded by a gene on chromosome 20 in a genomic region that has several genes with protease inhibitor domains [15] that has been implicated in regulating proteolysis. It is produced by macrophages and neutrophils and is known to inhibit the inflammatory response of these cells to various agents [16]. Elastase, a serine proteinase released in large quantities by neutrophils during inflammation, acts on PGRN to generate GRN peptides by cleaving short linker regions between the different GRN domains [14]. Interestingly, SLPI can inhibit this process by binding directly to the linker regions on PGRN as well as to elastase, thereby acting as a potent regulator of PGRN proteolytic processing.

The role of elastase and SLPI in regulating inflammation can be recognized by the contrasting effects of PGRN and GRN on epithelial cells and neutrophils. In cell culture, GRN(B) stimulates epithelial cells to secrete interleukin-8 (IL-8), a major chemoattractant for neutrophils and monocytes, whereas PGRN has no such effect. In response to proinflammatory cytokines, such as tumor necrosis factor alpha (TNFα), neutrophils adhere, spread, undergo degranulation and release a range of reactive species (including elastase) during respiratory burst. PGRN inhibits spreading, degranulation and respiratory burst of TNFα-activated neutrophils. In contrast, GRN (A) and GRN (B) peptides have no inhibitory effect. These observations implicate pro-inflammatory and anti-inflammatory roles for GRN and PGRN, respectively.

Although PGRN can inhibit TNFα-mediated neutrophil activation, it has no effect on neutrophils already undergoing a respiratory burst. This phenomena has also been seen when using reagents that disrupt signaling events that lead to the respiratory burst [17,18], suggesting PGRN somehow changes intracellular signaling events after TNFα binds to one of the TNF receptors [14]. In light of this, it is not surprising that wounds in SLPI deficient mice have increased leukocyte infiltration and elevated elastase activity, as well as impaired wound healing [19]. Addition of recombinant SLPI or PGRN to these animals normalizes wound response [14], suggesting that PGRN was being converted into GRNs as a result of unregulated proteolysis by elastase in wounds of SLPI-deficient mice. Deficiency in SLPI has the potential to disrupt the ratio of PGRN to GRNs resulting in excess GRNs and a net pro-inflammatory response. Collectively, these results suggest that activities of elastase (generating GRNs) and SLPI (inhibiting PGRN cleavage) are biological regulators of the innate immune response during wound healing.

Cytokines and hormones act as regulators of PGRN expression. Proinflammatory cytokines of the innate immune system, interleukin1 beta (IL-1β) and TNFα, activate *PGRN *gene expression in murine embryo fibroblasts [20]. The promoter for both human [21] and murine [22]*PGRN *contain regulatory elements that are involved in cytokine and growth-factor – regulated gene expression, including IL-6 response factor [21]. There is also increased expression of *PGRN *in inflammatory and immune disorders, such as rheumatoid arthritis [23], a zebra fish model of chronic tuberculosis [24] and a murine model simulating chemokine-induced alveolar monocyte trafficking [25]. PGRN is preferentially associated with cells of the innate immune system, including macrophages and neutrophils. IL-4, which is an anti-inflammatory cytokine of the adaptive immune system, decreases PGRN expression in certain myeloid cells [11]. Together these studies suggest a pleiotropic role for PGRN during inflammation in peripheral tissues.

## PGRN in the CNS

### Gene expression in the CNS

**Figure 2:**
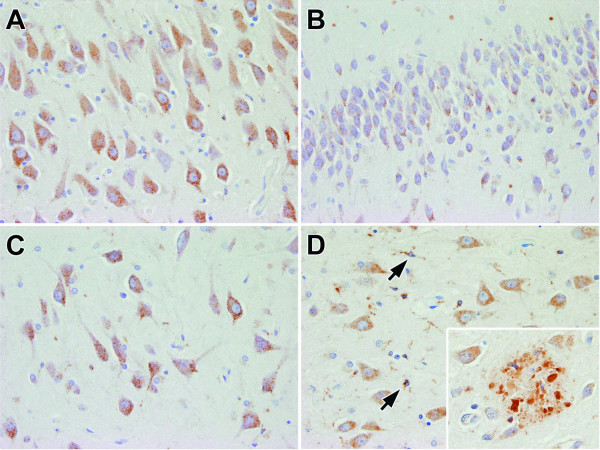
Immunohistochemical staining of human brain tissue using a PGRN-specific polyclonal antibody. In a neurologically normal individual, PGRN immunoreactivity is present in hippocampal pyramidal neurons, but particularly high in CA1 (A), dentate fascia (B) and endplate/CA4 (C). In AD, neurons and activated microglia (arrows) in the endplate are labeled (D), along with PGRN immunoreactivity associated with dystrophic neurites in senile plaques (inset).

Few studies have investigated the expression and function of PGRN in the CNS. Initial studies analyzing brain homogenates by northern blot analysis reported relatively low levels of expression of *PGRN *mRNA in the brain [7]. Using *in situ *hybridization techniques in adult rodent brains, *PGRN *mRNA was found to be abundant in specific neuronal subsets, including cortical pyramidal neurons, cerebellar Purkinje cells and pyramidal neurons of the hippocampus [6]. Immunohistochemical studies have also shown expression of PGRN in certain neuronal populations (Figure 2) [26,27]. The subcellular location of PGRN in neurons is currently unknown, but preliminary studies suggest that it may be associated with endosomal or lysosomal vacuoles.

During CNS development [2]*PGRN *expression is high in neuroepithelial cells in the embryo and then decreases in fetal development, where it is restricted to the forebrain, olfactory lobes, retinal ganglion and spinal cord. Later, *PGRN *is expressed throughout the neocortex, but not in regions where neurogenesis is known to occur, such as the subventricular zone [28]. The differential expression of *PGRN *in specific cell types during forebrain development suggests a role in the developing CNS.

### Neurotrophic properties of PGRN

PGRN promotes growth of PC12 cells, a pheochromocytoma-derived neuronal cell line that responds poorly to most nerve growth factors [6]. The only other growth factors shown to have an effect on PC12 cells similar to PGRN are insulin growth factors-1 and 2 (IGF-1 and IGF-2). In embryonic fibroblasts PGRN activates similar signal transduction pathways as IGF-1 and IGF-2. [29]. Although these results suggest a neurotrophic role for PGRN, there are subtle, but potentially important, differences between PGRN and other growth factors. For example, in a blunt-force traumatic brain injury model in mice, most growth factors, such as neuregulin and brain derived neurotrophic factor, show robust increases in as few as three hours. In the same model, increases in PGRN mRNA do not occur until 24 hours, by which time the expression of the other growth factors have returned to normal [30]. The delayed induction of PGRN and its potential roles in normal and pathological conditions requires further investigation, but the available evidence suggests that PGRN may be important in long-term neuronal survival, but not a significant factor in response to acute neuronal injuries.

### Microglial expression of PGRN

Non-neuronal cell types also show evidence of *PGRN *expression in the CNS. Although initial *in situ *hybridization studies of *PGRN *expression did not detect any signal in glial cells [6], more recent immunohistochemical studies have shown strong immunoreactivity in microglia [26,27,31] (Figure 2), especially when activated. Microglia are intrinsic CNS glial cells derived from the mononuclear phagocyte system [32,33], which fits with the fact that PGRN is a protein produced by hematopoietic cell types [6,7,11]. In contrast, astrocytes and oligodendroglia, which are derived from the neural tube, have no or very low levels of PGRN immunoreactivity [27].

Microglia represent about 5–20% of all CNS glia [32,33]. Current evidence suggests that microglia are derived from monocytes that enter the developing brain during embryogenesis, after which they differentiate into resident microglia [34]. There is little turnover of resident microglia, but more so for perivascular macrophages [35]. Under normal conditions microglia are quiescent and characterized morphologically by ramified processes and a small soma. In response to pathologic insults, such as traumatic injury, infection or neurodegeneration, microglia become activated. In the activated state microglia have proliferative potential, and they undergo migration and phagocytosis [36,37]. During injury-induced microglial activation, increases in microglia occurs through both mitosis of resident microglia [38] and migration of bone-marrow derived cells into the CNS [39-41].

Microglial activation is associated with changes in shape and functional properties, including increases in cell surface molecules (e.g., HLA-DR and β2-integrins) and production of proinflammatory cytokines (e.g., IL-1β and TNFα), chemokines, growth factors and inflammatory mediators (e.g., platelet activation factor) [42]. In most conditions, microglial activation is accompanied by reactive astrocytic gliosis [43]. Together these features are the face of neuroinflammation, the major response of the innate immune system in the CNS.

**Figure 3:**
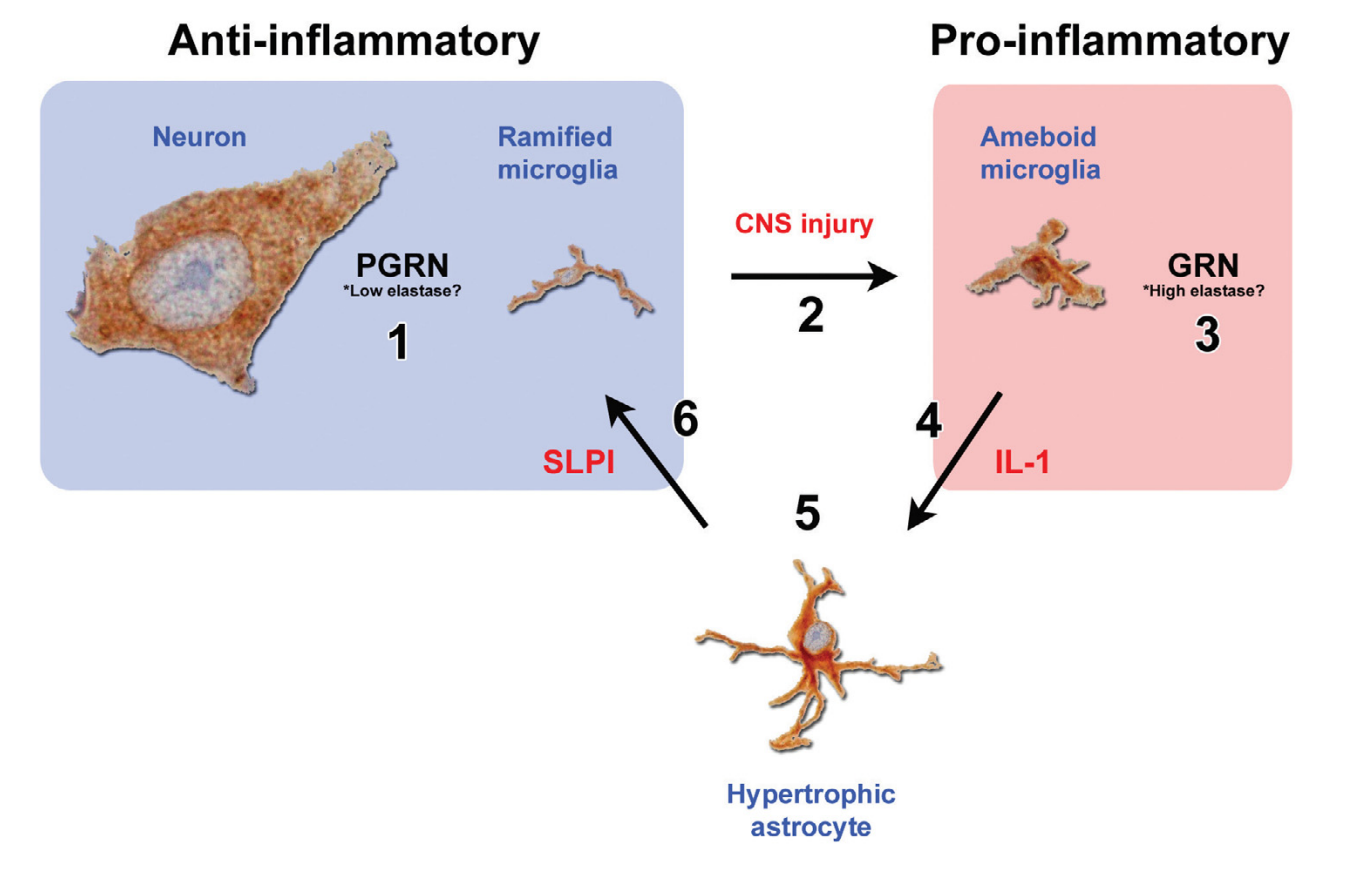
Hypothetical interaction between PGRN, elastase and SLPI in the CNS. **1**) PGRN expression in neurons in the absence of microglial-derived elastase has potentially growth factor and anti-inflammatory properties. **2**) In response to CNS injury, microglia are activated and release inflammatory mediators, including proteases. **3**) PGRN and elastase levels increase, resulting in the proteolytic cleavage of PGRN into GRNs, which may contribute to the inflammatory milieu. **4**) Inflammatory signals such as IL-1β derived from activated microglia cause changes in nearby cells. **5**) Astrocytes become reactive in response to inflammatory stimulus from activated microglia. **6**) Reactive astrocytes produce SLPI, which along with its other anti-inflammatory properties, inhibits the proteolytic cleavage of PGRN into GRN as a means of feedback regulation of the inflammatory response.

In the context of neurodegenerative diseases, sustained microglial activation has been linked to neuronal injury and loss, in part mediated by excessive production of proinflammatory cytokines and other toxic species [44-46]. According to this theory, neuronal injury occurs through a "by-stander" mechanism [47]. Considering the role of PGRN during inflammation in the periphery, along with the mechanism by which microglia respond, tight regulation of *PGRN *expression in the CNS would seem important. A model of ischemic stroke in rats [48] provides a theoretical means by which PGRN may be regulated during CNS inflammation (Figure 3). Microglia and astrocytes both respond to hypoxic-ischemic neuronal injury with abundant cross-talk between these cell populations. For example, IL-1β is produced by activated microglia and is a major activator of astrocytes [49]. Conversely, colony stimulating factor 1 (CSF-1) and granulocyte-macrophage colony stimulating factor are potent microglial growth factors that are produced by activated astrocytes [50,51]. Interestingly, astrocytes also express SLPI, the elastase- and PGRN-binding protein implicated as a regulator of PGRN proteolysis in the periphery [14], during ischemic stroke where it reduces ischemic-induced injury [48]. It remains to be shown whether SLPI regulates PGRN proteolysis in the CNS. As noted above, in the periphery the relative balance between the activities of elastase and SLPI influences the levels of the anti-inflammatory PGRN and the pro-inflammatory GRNs. It is worth noting that cultured microglia have been shown to produce elastase [52], which is also thought to be a key player in this inflammatory switch mechanism. Environmental or genetic factors that may affect the normal regulation of PGRN could have adverse consequences leading to neuroinflammation and neurodegeneration.

Although the neurotoxic potential of chronically activated microglia has been the focus of most studies, a growing body of research suggests that microglia may play a neuroprotective role, as well [53]. This has prompted some researchers to suggest that the loss of normal physiologic functions of microglia may contribute to neurodegeneration. Since PGRN has been shown to have trophic functions in the periphery, and it is expressed by activated microglia, it is intriguing to speculate that microglia-derived PGRN may support neuronal viability or possibly perform a role equivalent to wound healing in the periphery mediated by its neurotrophic activity. There are several lines of evidence that lend support to this hypothesis. First, microglia have the ability to produce neurotrophic factors, such as thrombospondin [53,54]. Second, microglia produce other growth factors, such as TGF-β, during injury and repair [55,56]. Third, as mentioned previously, PGRN has growth factor properties on neuronal cells in culture [6]. Obviously, much remains to be learned about potential neurotrophic properties of microglia-derived PGRN as it relates to neurodegeneration.

## PGRN in CNS disorders and animal models of CNS disease

Much of the available information on *PGRN *expression in various disorders comes from unbiased expression array studies in which *PGRN *was shown to be one of the responsive genes increased compared to controls. Interestingly, almost all the mRNA expression studies that have shown differential expression of *PGRN *share the common property of microglial activation and inflammation, leading some researchers to speculate that the increase in PGRN expression is closely related to microglial activation and neuroinflammation [1].

### PGRN in models of CNS viral infection

PGRN has been shown to be increased in young mice during the host response to two different strains of Sindbis virus with varying neurovirulence [57]. Virus replication, viral burden and evidence of apoptosis were greater with the more virulent strain, even though neuronal cell tropism was the same. Histologic evidence of inflammation was mild, but the gene expression profile highlighted differences between virulent and non-virulent strains. In particular, a number of chemokine genes, as well as *PGRN*, that are up-regulated in microglia during inflammation were increased during infection by the virulent strain. Given that both microglia and neurons expressed *PGRN*, it is not possible to know the cellular origin of the *PGRN *in this and other models.

### PGRN in models of Creutzfeldt-Jakob disease (CJD)

Studies of the gene expression profile of microglial cell cultures taken from mice infected with CJD (M-CJD) have provided some information on the microglial expression of PGRN in response to different activating stimuli [58]. The pathogenic prion protein has been shown to accumulate in activated microglia, although the mechanism for this remains to be determined [59]. Proinflammatory transcripts, such as IL-1β and complement factors, as well as PGRN were increased in M-CJD. When normal microglia were challenged with endotoxin (LPS) and interferon-gamma (IFNγ), to mimic the expression profile of activated microglia, PGRN expression was substantially suppressed compared to that seen in M-CJD. In addition, both IL1β and TNFα were highly expressed in M-CJD and LPS treated mice, but PGRN was only increased in MCJD. This paradoxical response of PGRN may relate to the pleiotropic functional properties of PGRN not expected with more traditional growth factors; PGRN may function as a growth factor or anti-inflammatory agent as an intact molecule, but as a source of diverse inflammatory mediators, when it undergoes proteolysis to GRNs. The only other transcripts that had an expression pattern similar to PGRN were LY5, leukocytes common antigen and CD84, which are involved in inflammation and intracellular communication [58]. These particular studies suggest that microglial expression of PGRN increases during neuroinflammation in neurodegenerative disease, but not during simple microglial induction through LPS treatment. As a result, fundamental questions need to be addressed concerning the role of PGRN in microglial function. In particular, it is not known if *PGRN *is differentially expressed by intrinsic microglia or by bone-marrow derived macrophages.

### PGRN in models of lysosomal storage disease

Mucopolysaccharidoses (MPS) type I and type IIIB are lysosomal storage diseases that affect the CNS. Mouse models have suggested that activated microglia accelerate the neuronal degeneration caused by lysosomal storage [60]. Microarray gene analysis of cortical brain tissue from these mouse models showed a prominent inflammatory pattern of gene expression, which was accompanied by increases in *PGRN *[61]. They also demonstrated that microglia in these mice contain massive lysosomal vacuoles. It remains to be determined if the increase in PGRN in these models is due to neuronal degeneration or to microglial activation in response to abnormal lysosomal storage.

### PGRN in ALS

*PGRN *mRNA expression in ALS spinal cord has been found to be increased by 400% compared with controls [62]. This is likely related to microglial activation, which has been implicated in the pathogenesis of ALS [63]. As discussed later, the neuronal cytoplasmic inclusions in motor neurons of ALS are composed of the same protein (TDP-43) as those in FTLD-U [64,65], which suggests a fundamental linkage between ALS and FTLD.

### PGRN in Alzheimer's disease (AD)

Several recent studies have shown PGRN immunoreactivity in AD is associated with amyloid plaques [31] (Figure 2), including labeling of microglia and dystrophic neurites [27]. Plaque-related dystrophic neurites are large axonal varicosities that are associated with reduced dendritic spine density and shaft diameter [66]. Considering that *PGRN *expression is increased during injury and repair in the periphery, the presence of PGRN immunoreactivity in dystrophic neurites could reflect a reparative response in damaged axons. Complementing this hypothesis is the observation that dystrophic neurites are constantly being formed and resolved; however, the rate of dystrophic neurite formation exceeds their resolution [66]. Considering the growth factor properties of PGRN in the periphery, it is tempting to speculate that PGRN may be involved in neuritic remodeling.

Most studies have shown PGRN to be present in the perikarya of neurons, but it is unknown whether PGRN is also normally present in axons or dendrites. If PGRN is normally present in axons, then the accumulation of PGRN in dystrophic neurites may also reflect disruption of axonal transport, similar to what is found for other axonally transported proteins such as amyloid precursor protein [67].

## PGRN in FTLD-U

### Mutations in PGRN cause FTLD-U

Recent interest in PGRN has been fueled by the discovery of mutations in *PGRN *in some families with autosomal dominant FTLD-U [27,31,68-70]. Prior to this discovery, no mutations in *PGRN *had been associated with any human disorder [1]. FTLD-U is a member of a diverse group of neurodegenerative disorders that produce frontotemporal dementia, which accounts for 5–15% of all dementia disorders [71]. The clinical phenotype in patients with *PGRN *mutations is similar to those with sporadic frontotemporal dementia. They have a variable age of onset, and the dementia tends to be characterized by prominent behavioral and language dysfunction, usually a progressive non-fluent aphasia [72]. Mild parkinsonism is common, but motor neuron disease is usually absent [26,73].

Mutations in the gene for the microtubule associated protein tau (*MAPT*) on chromosome 17q21, which incidentally is in close proximity to the *PGRN *gene, are known to be responsible for 10–20% of familial frontotemporal dementia [74]. These cases are pathologically characterized by the abnormal accumulation of hyperphosphorylated tau protein in neurons and glia, which is distinct from the FTLD-U pathology in *PGRN*-related frontotemporal dementia. To date no *MAPT *mutations have been detected in FTLD-U [75].

A recent study reported the frequency of *PGRN *mutations in FTLD to be 5% in a patient referral series, which is a similar frequency to that of *MAPT *mutations in the same series of patients [69]. At least 35 different pathogenic *PGRN *mutations have now been identified, all of which are predicted to create functional null alleles with the majority causing premature termination of the coding sequence [69]. The introduction of a premature termination codon results in degradation of the mutant mRNA species by nonsense mediated decay [31]. As a result, there is no production of mutant protein. It therefore appears that *PGRN *mutations cause FTLD-U due to a partial loss of functional PGRN, (haploinsufficiency), rather than accumulation of mutant protein characteristic of frontotemporal dementia due to *MAPT *mutations.

Several other genes or chromosomal loci have been identified for FTLD-U, including mutations in the gene for valosin-containing protein (VCP) [76] and CHMP2b (charged multivesicular body protein 2b) [77]. VCP is an endoplasmic reticulum-associated protein that is involved in ER-stress related protein degradation [78,79]. While little is known about CHMP2b, it appears to play a role in endosomal trafficking through the ESCRT (endosomal secretory complex required for trafficking) III complex, which may be involved in degradation of growth factors [80]. Given the preliminary evidence that PGRN is a neurotrophic factor that may be associated with endosomal or lysosomal vacuoles, it raises the intriguing possibility that the three major genes for FTLD-U are associated with defects in protein degradation linked to membranous cytoplasmic organelles.

**Figure 4:**
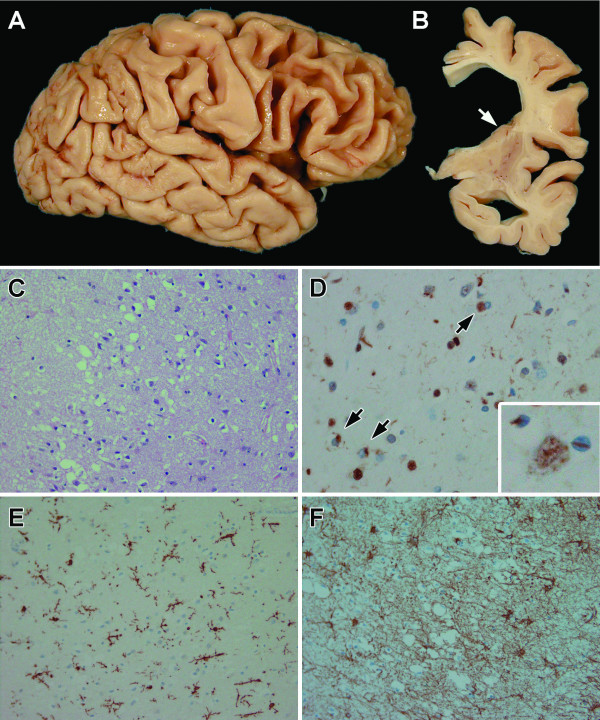
Neuropathology of FTLD-U with PGRN mutations. Gross cortical atrophy is visible in frontal and superior temporal lobes (A). In coronal sections (B), the lateral ventricle is dilated and the caudate nucleus is flat (arrow). Laminar spongiosis in the layer II of the cortical ribbon (C) is associated with TDP-43-positive neuronal cytoplasmic (D, arrows) and "lentiform" intranuclear inclusions (inset). Severe neuronal loss (D) in these regions is associated with microgliosis (E) and astrogliosis (F), shown by a microglial marker [ionized calcium-binding adapter molecule 1 (Iba-1)) and glial fibrillary acidic protein (GFAP) specific immunohistochemistry, respectively.

FTLD-U is the most common pathology associated with frontotemporal dementia and is characterized by focal cortical atrophy with spongiosis, gliosis and ubiquitin-immunoreactive neuronal cytoplasmic inclusions (NCI) and neurites in layer II of affected cortices and in the hippocampal dentate fascia [81] (Figure 4). Most cases also have neuronal loss in the hippocampus consistent with hippocampal sclerosis [82]. In postmortem series, pathology was more severe and associated with marked neuronal loss and gliosis in cases with *PGRN *mutations compared to those without mutations [73]. The presence of lentiform shaped neuronal intranuclear inclusions (NII) is a consistent feature of cases with *PGRN *mutations, but is not entirely specific [83,84] (Figure 4).

As in any neurodegenerative disorder neuronal loss in FTLD-U is accompanied by reactive astrogliosis and microglial activation (Figure 4). There are no studies of microglial functional properties in FTLD-U cases with and without *PGRN *mutations. A closer look at the inflammatory response in cases with and without the *PGRN *mutation would also be helpful in understanding the biological role of *PGRN *in FTLD-U.

### Neuronal inclusions in FTLD-U contain TDP-43

Consistent with the haploinsufficiency mechanism, NCI and NII in familial FTLD-U with *PGRN *mutations do not contain mutant PGRN [26]. A major component of the inclusion bodies was recently shown to be TAR DNA binding protein-43 (TDP-43) [64,65] (Figure 4). The same protein was also found to be present in the neuronal inclusions in ALS. Very little is known about the biological function of TDP-43. Originally identified as a ubiquitously expressed 43-kDa protein, TDP-43 binds to the TAR DNA in the long terminal repeat region of human immunodeficiency virus (HIV)-1, resulting in repression of promoter activity [85]. It was also independently identified as a regulator of alternative splicing of exon 9 of the cystic fibrosis transmembrane conductance regulator transcript, through its ability to bind (UG)_n_-repeated RNA sequences [86]. It is thought that these DNA and RNA binding properties implicate TDP-43 as a transcription regulator through one of its two RNA-recognition motifs [85,87]. TDP-43 is widely expressed in many tissues, including the brain [85], where immunohistochemistry highlights diffuse, but grainy expression of nuclei of neurons and other CNS cells. In FTLD-U, the normal nuclear staining pattern is absent in neurons that contain NCI and NII, leading investigators to suggest that TDP-43 is translocated from the nucleus to the cytoplasm [64] or that TDP-43 is prevented from crossing the nuclear membrane possibly, as a result of hyperphosphorylation of TD-43 [88]. This abnormal metabolism of TDP-43 in the FTLD-U seems central to the disease pathogenesis given that the gene encoding TDP-43 is highly conserved among different species [89] consistent with an essential, yet currently unknown biological function.

### TDP-43 and PGRN

**Figure 5:**
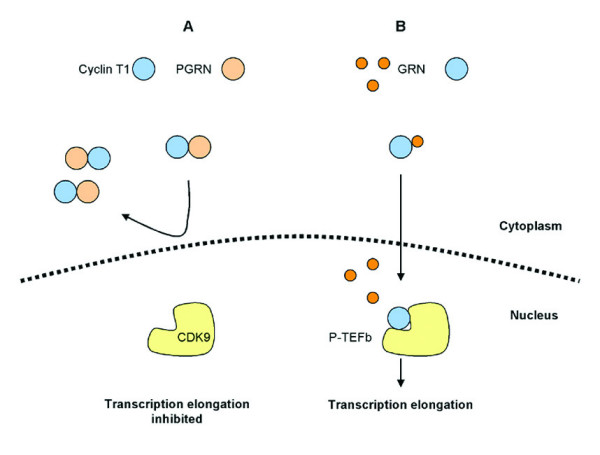
Schematic model of nuclear trafficking of cyclin T1 and it alterations by PGRN and GRN. Cyclin T1 binds PGRN and some of its GRN derivatives when co-expressed in COS7 cells [91]. **A**) When cyclin T1 is expressed with full length PGRN, both proteins are localized in the cytoplasm. **B**) In contrast, when expressed with the C-terminal GRN, CDE, cyclin T1 and GRN are found in the nucleus, enabling down-stream transcription elongation. Given the evidence that PGRN and GRN are associated with translocation of proteins such as cyclin T1 from cytoplasm to nucleus, it is tempting to hypothesize that PGRN might be involved in similar regulation of TDP-43 nuclear-cytoplasmic translocation. With decreased functional PGRN in FTLD-U associated with *PGRN *mutations, TDP-43 translocation might be perturbed, leading to accumulation in the cytoplasm and formation of NCI.

The relationship between PGRN and TDP-43 and their respective roles in neurodegeneration is currently unknown. Although most of the biological considerations in this review have focused on PGRN as a secreted protein; there is data suggesting an intracellular and possibly even an intra-nuclear role for PGRN. While most growth factors function through binding to cell surface receptors with subsequent intracellular signaling, there is recent evidence to suggest that some growth factors or inflammatory mediators may cross the cell membrane through currently poorly defined means to gain access to the cytosol and even the nucleus (reviewed in [90]). It is intriguing to speculate that PGRN may be involved in intracellular trafficking. There is little experimental evidence to support this hypothesis at present, but studies of transcriptional regulation hint at a possible link between PGRN and TDP-43. In these studies intracellular trafficking of certain proteins has been shown to be modulated differentially by PGRN and GRN. PGRN and one of the GRN peptides, CDE, have the ability to bind cyclin T1 [91], an essential protein component of the positive transcription elongation factor needed to phosphorylate the largest subunit of RNA polymerase II, resulting in transcriptional elongation (reviewed by [92]). When PGRN and cyclin T1 are co-expressed in the same cell, both are restricted to cytoplasm, thereby inhibiting transcription elongation [91]. In contrast, when co-expressed with GRN (CDE), both proteins are localized mainly in the nucleus. Given these results and the abnormal location of TDP-43 in the cytoplasm rather than the nucleus in FTLDU, it raises the possibility that PGRN might be involved in the trafficking of TDP-43. Interestingly, PGRN is known to bind HIV-1 and HIV-2 tat proteins [91,93]. Tat proteins associate with TAR DNA, a property that is also shared by TDP-43 [85]. If TDP-43 has PGRN-binding properties, coupled with what is known about the effects of PGRN and GRN on nuclear-cytoplasmic trafficking of certain proteins, one can envisage how dysfunction or dysregulation of PGRN might contribute to abnormal compartmentalization of TDP-43 (Figure 5).

## Conclusion

PGRN is a complex protein that has distinct functional properties as an intact precursor protein compared to GRN peptides derived from its proteolytic cleavage. In peripheral tissues, it has been implicated in development, maintenance, repair, inflammation and neoplasia. The interactions of elastase, SLPI and PGRN leads to speculation that PGRN may have pro- or anti-inflammatory properties depending upon the extent of regulated proteolysis of PGRN and generation of pro-inflammatory GRN peptides.

PGRN is expressed in the developing CNS, where its growth promoting function has been suggested. In the mature CNS immunohistochemical and *in situ *hybridization studies have shown that certain populations of neurons express PGRN. There is evidence that full-length PGRN may function as a neuronal growth factor. In this light, pathogenic *PGRN *mutations that lead to decreases in functional PGRN may produce neurodegeneration in FTLDU as a result of decreases in neurotrophic activity.

Microglia are the other major cell type that expresses *PGRN *in the CNS. Trauma, infection and neurodegeneration are all accompanied by increases in *PGRN *mRNA expression. These results are consistent with the notion that *PGRN *expression is directly or indirectly related to microglial proliferation and activation, implicating PGRN in neuroinflammation and potentially brain repair. The role of altered PGRN in microglia in this regard needs further investigation since some studies have suggested trophic rather than toxic functions of microglia in specific circumstances. Studies of microglia and the inflammatory responses in *PGRN *mutation carriers and controls, as well as in *PGRN *knock out mice will be useful in determining not only the normal function of PGRN in the CNS, but also its role in the pathogenesis of FTLDU.

## Competing interests

The author(s) declare that they have no competing interests.

## Authors' contributions

ZA wrote the initial draft and produced the hypothetical mechanisms and pathological images. Modification suggested by IM, MH and DD were applied by ZA and DD to the final draft. MH and IM made particular contributions to the sections on the genetics and pathology of FTLD-U, respectively. All authors read and approved the final version.
